# Gender differences in demographic and clinical characteristics in patients with HBV-related liver diseases in China

**DOI:** 10.7717/peerj.13828

**Published:** 2022-08-05

**Authors:** Mei Liu, Lu Li, Jing Zhao, Gabor S. Ungvari, Chee H. Ng, Zhongping Duan, Su-Jun Zheng, Yu-Tao Xiang

**Affiliations:** 1 Artificial Liver Center, Beijing YouAn Hospital, Capital Medical University, Beijing, China; 2 The Affiliated Brain Hospital of Guangzhou Medical University (Guangzhou Huiai Hospital), Guangzhou, China; 3 University of Notre Dame Australia, Fremantle, Australia; 4 Division of Psychiatry, School of Medicine, University of Western Australia/Graylands Hospital, Perth, Australia; 5 Department of Psychiatry, The Melbourne Clinic and St Vincent’s Hospital, University of Melbourne, Richmond, Victoria, Australia; 6 Centre for Cognitive and Brain Sciences, University of Macau, Macao SAR, China; 7 Institute of Advanced Studies in Humanities and Social Sciences, University of Macau, Macao SAR, China; 8 Unit of Psychiatry, Department of Public Health and Medicinal Administration, & Institute of Translational Medicine, Faculty of Health Sciences, University of Macau, Macao SAR, China

**Keywords:** Gender difference, HBV-related liver diseases, Demographic

## Abstract

**Background:**

The gender differences in demographic and clinical characteristics were examined in patients with hepatitis B virus (HBV)-related liver diseases.

**Methods:**

Overall, 634 patients (44.7 ± 13.8 years) were consecutively included. Data of demographic and clinical characteristics were collected during an assessment interview. Comparisons between male and female patients in terms of demographic and clinical data were carried out using univariate analyses. The independent associations between the demographic and clinical variables and gender were examined with either logistic regression or analysis of covariance as appropriate.

**Results:**

The study sample consisted of 452 male and 182 female patients. Multiple logistic regression analyses revealed that being employed (OR = 3.4), personal monthly income <3,000 yuan (OR = 0.3), being current alcohol users (OR = 6.4), Cirrhosis (OR = 5.9), Hepatocellular Carcinoma (HCC) (OR = 8.5) and having less severe insomnia (OR = 0.6) were independently associated with male gender. The analysis of covariance revealed that after controlling for other potential confounding variables, later onset of HBV-related diseases (*F* = 4.5, *p* = 0.03) and older age (*F* = 6.7, *p* = 0.009) were independently associated with male gender.

**Conclusions:**

Given the significant clinical differences in male and female patients with HBV-related liver diseases, more attention should be given to gender-specific treatment and prevention for this population.

## Introduction

Hepatitis B virus (HBV) infection is a serious infectious disease. The WHO estimated around 400 million persons suffer from chronic HBV infection worldwide (World Health Organization, 2021). Previous studies found that there are 93 million HBV carriers in China, of which 30 million have chronic hepatitis B (CHB) (Lyu et al., 2016). HBV is a risk factor for hepatocellular carcinoma (HCC) and liver failure, associated with significant personal suffering and large economic and healthcare burden (Lu et al., 2013).

Current evidence suggests that there are important gender differences in terms of the epidemiology and clinical features of HBV-infections. For instance, chronic HBV infection is more common in males compared to in females (10.7% *vs* 4.4%) among those who receive vaccination at birth and are followed up for over 18 years (Su et al., 2007). HBV-related HCC is much more common in men compared to women, with a ratio of 5–7:1 (Lee et al., 1999). The prevalence of HBV-related liver diseases and liver disease-related death are much higher in males compared to females (Shen et al., 2011). Compared to females, males are more likely to progress to severe liver diseases (Lyu et al., 2016; Stroffolini et al., 2015). One study (Stroffolini et al., 2015) that investigated the gender differences in chronic Hepatitis B surface antigen (HBsAg) carriers in Italy found that male gender was related to more severe liver diseases. Baig (2009) found male gender and age >50 years were associated with increased risk of HCC.

To improve the prognosis of HBV-infected patients, it is essential to understand the gender differences in terms of clinical features. To date, some studies has determined the gender differences in demographic, physical and psychosocial characteristics in HBV-infected patients, including lifestyle (*e.g.*, alcohol consumption, cigarette smoking) (Lyu et al., 2016) and social behaviors, which are associated with the occurrence of HBV-related liver diseases. For instance, previous studies found that the higher prevalence of alcohol drinking (10.3% *vs.* 1.6%) and smoking (15.7% *vs.* 4.8%) among men could be partly attributed to the gender disparity in liver diseases (Cao et al., 2021). The gender differences in epidemiology and clinical features of HBV-associated liver diseases could be largely attributed to differences in sex hormones, immune regulation, susceptibility and exposure to HBV infections between males and females (Anna & Walter, 2015; Guy & Peters, 2013).

Although China has the highest number of HBV-infected patients worldwide, no published studies in China have examined the gender differences in clinical characteristics. Hence, this study compared the gender differences in the demographic and clinical data of Chinese patients with HBV-related liver diseases.

## Materials & Methods

### Participants

This study was carried out in a teaching hospital of Capital Medical University for infectious diseases. Outpatients or hospitalized patients were invited to participate in this study using consecutive sampling method based on the following inclusion criteria: (1) aged 18 years and older; (2) diagnosed as HBV carrier, CHB, hepatitis B cirrhosis or HCC (Jia & Li, 2011); (3) had an ability to understand the contents of the assessment. Beijing YouAn Hospital Clinical Research Ethics committee approved this study. Written informed consent was provided by patients.

### Measures

All patients were assessed by two research physicians. Basic socio-demographic and clinical characteristics, such as gender, age, education, treatment status (*e.g.*, in- or outpatient), marital status, employment status, current alcohol use, being local residents, living alone, personal income, family history of psychiatric disorders, and chronic physical diseases, were collected using a standard data collection form.

Major depression was diagnosed in a clinical interview conducted by research physicians who were trained in use of the Mini International Neuropsychiatric Interview (MINI), Version 5.0, Chinese version (Sheehan et al., 1998). Overall psychosocial functioning was assessed with Global Assessment of Functioning (GAF) (Startup, Jackson & Bendix, 2002), with lower scores reflecting poorer functioning. A person who drank alcoholic beverage at least once each month in past year was defined as a current alcohol user.

The three types of insomnia symptoms in the past month (Liu & Zhou, 2002) were assessed with three standard questions: “*Do you have difficulties in falling sleep?*” for difficulty initiating sleep; “*Do you have the difficulties in maintaining sleep and wake up often?*” for difficulty maintaining sleep; and for early morning awakening “*Do you wake up in the midnight or early morning and have difficulties in falling sleep again*?” If patients answered “often” to at least one of the three questions, they were classified as “having insomnia”.

### Data analysis

Gender difference in demographic and clinical variables were compared using chi-square tests, *t*-tests and Mann–Whitney *U* test, as appropriate. The independent associations between the variables that significantly associated with gender in the above univariate analyses and gender was examined with either logistic regression (for categorical variables) or analysis of covariance (ANCOVA for continuous variables) after controlling for the other potential confounding variables that were significant between the two genders. The level of significance was set at 0.05 (two-tailed). Due to collinearity between the treatment status (in-or outpatient), diagnosis of HBV-related liver diseases and age of onset of HBV, the treatment status (in-or outpatient) were not entered in the logistic regression model. The Hosmer and Lemeshow test and Pearson’s chi-square test were used to estimate the Goodness-of-Fit of the model in the binary logistic regression and multinomial logistic regression analyses, respectively.

## Result

Overall, 812 patients with HBV-related diseases (including Carrier, CHB, Cirrhosis, HCC) were invited; 634 patients (452 male and 182 female) fulfilled the study criteria, giving a participation rate of 78.1%.

Table 1 presents the frequency of HBV-related liver diseases by gender and shows the ratio by age group. The predominance of male gender ratio existed in all age groups. The male to female ratio was maximum between the age groups of 41–45 (4.7:1) and 46–50 (4.9:1), while the ratio was minimum between the age group of 18–30 (1.5:1–1.6:1) and above 66 years (1.8:1).

**Table 1:** The frequency of hepatitis B-related diseases in males and females (*n* = 634).

Variable	Female (*n* = 182)		Male (*n* = 452)		Male:Female	Total (*n* = 634)	
Age (years)	*N*	%	*N*	%	Ratio	*N*	%
18–25	14	7.7	21	4.6	1.5:1	35	5.5
26–30	23	12.6	38	8.4	1.6:1	61	9.6
31–35	15	8.2	29	6.4	1.9:1	44	6.9
36–40	19	10.4	41	9.1	2.1:1	60	9.5
41–45	12	6.6	57	12.6	4.7:1	69	10.9
46–50	13	7.1	64	14.2	4.9:1	77	12.1
51–55	19	10.4	59	13.1	3.1:1	78	12.3
56–60	20	11.0	52	11.5	2.6:1	72	11.4
60–65	26	14.3	53	11.7	2.0:1	79	12.5
66 and above	21	11.5	38	8.4	1.8:1	59	9.3
							
HBV-related liver disease	*N*	%	*N*	%	Ratio	*N*	%
Carrier	27	14.8	33	7.3	1.2:1	60	0.09
Chronic HBV	80	44.0	149	32.9	1.8:1	229	0.36
HBV cirrhosis	42	23.1	139	30.8	3.3:1	181	0.29
Liver Cancer	33	18.1	131	29.0	3.9:1	164	0.26

Of the study sample, 54.4% were inpatients, and the mean age was 47.7 years. The mean ages of onset and duration of HBV-related liver diseases were 33.7 years and 14.0 years, respectively. The proportion of people who suffered from insomnia and major depression was 26.2% and 6.5%, respectively. The basic demographic and clinical data of patients by gender are shown in Table 2. Male patients were older (48.0 ± 13.2 *vs.* 46.9 ± 15.1; *p* < 0.001), more likely to be employed (97.3% *vs* 81.9%, *p* < 0.001), married (88.5% *vs* 82.4%, *p* = 0.04) and alcohol users (22.1% *vs* 4.9%, *p* < 0.001), had personal income >3,000 yuan (74.1% *vs* 48.9%, *p* < 0.001), had a later onset of HBV-related diseases (34.5 *vs* 31.9, *p* = 0.02), and more severe HBV-related liver diseases (*p* < 0.001). They were also less likely to have insomnia (23.2% *vs* 33.5%, *p* = 0.008), depression (4.9% *vs* 10.4%, *p* = 0.01) and suicidality (1.8% *vs* 7.7%, *p* < 0.001). In multivariate analyses, older age(*F* = 6.7, *P* = 0.009), later onset of HBV-related diseases (*F* = 4.5, *p* = 0.03), being employed (OR = 3.4, 95% CI [1.3–8.3], *p* = 0.008), had personal income <3000 yuan (OR = 0.3, 95% CI [0.2–0.5], *p* < 0.001), being current alcohol users (OR = 6.4, 95% CI [2.9–13.9], *p* < 0.001), having less insomnia (OR = 0.6, 95% CI [0.3–0.9], *p* = 0.03) and more severe hepatic cirrhosis (OR = 5.9, 95% CI [2.7–12.9], *p* < 0.001) and HCC (OR = 8.5, 95% CI [3.6–19.9], *p* < 0.001) were associated with male gender.

**Table 2:** Comparison of basic demographic and clinical characteristics between genders.

	Whole sample (*n* = 634)		Women (*n* = 182)		Men (*n* = 452)		Statistics						
							Univariate analyses			Multiple logistic regression analyses			Hosmer and Lemeshow test
	*N*	%	*N*	%	*N*	%	*χ* ^2^	**df**	***p***	**OR**	**95 CI%**	***p***	***p***
Inpatients	345	54.4	75	41.2	270	59.7	17.9	1	**<0.001**	–	–	–	–
Married	550	86.8	150	82.4	400	88.5	4.1	1	**0.04**	1.4	0.8–2.6	0.1^b^	**0.001**
Employed	589	92.9	149	81.9	440	97.3	47.1	1	**<0.001**	3.4	1.3–8.3	**0.008** ^c^	0.9
Local residents	272	42.9	76	41.8	196	43.4	0.1	1	0.1	–	–	–	–
Living alone	22	3.5	8	4.4	14	3.1	0.6	1	0.4	–	–	–	–
Personal income <3000 yuan	210	33.1	93	51.1	117	25.9	37.2	1	**<0.001**	0.3	0.2–0.5	**<0.001** ^d^	0.83
Having health insurance	14	2.2	2	1.1	12	2.7	0.8	1	0.3	–	–	–	–
Family history of psychiatric disorders	17	2.7	6	3.3	11	2.4	0.1	1	0.7	–	–	–	–
Current alcohol use	109	17.2	9	4.9	100	22.1	26.8	1	**<0.001**	6.4	2.9-13.9	**<0.001** ^e^	0.81
Chronic physical diseases	282	44.5	79	43.4	203	44.9	0.1	1	0.7	–	–	–	–
Insomnia	166	26.2	61	33.5	105	23.2	7.1	1	**0.008**	0.6	0.3–0.9	**0.03** ^f^	0.97
HBV-related liver disease							20.7	3	**<0.001**	–	–	**–**	**<0.001** ^l^
Carrier	60	9.5	27	14.8	33	7.3					1		
CHB	229	36.1	80	44.0	149	33.0				1.7	0.9–3.4	0.08^g^	–
Cirrhosis	181	28.5	42	23.1	139	30.8				5.9	2.7–12.9	**<0.001** ^g^	–
HCC	164	25.9	33	18.1	131	29.0				8.5	3.6–19.9	**<0.001** ^g^	–
Major depression	41	6.5	19	10.4	22	4.9	6.6	1	**0.01**	0.8	0.2–2,5	0.7^h^	–
suicidality	22	3.5	14	7.7	8	1.8	13.5	1	**<0.001**	0.3	0.05–2.0	0.2^i^	–
							Univariate analyses			ANCOVA			
	Mean	SD	Mean	SD	Mean	SD	**T / Z**	**df**	***p***	***F***	**df**	***p***	
Age (years)	47.7	13.8	46.9	15.1	48.0	13.2	−0.9	632	**<0.001**	6.7	–	**0.009** ^j^	–
Age of onset of HBV (years)	33.7	14.3	31.9	14.9	34.5	14.0	−2.2	–^a^	**0.02**	4.5	–	**0.03** ^k^	–
Duration of HBV-related liver disease (years)	14.0	11.2	14.6	11.3	13.8	11.2	−1.0	–^a^	0.29	–	–	–	–
Education (years)	11.0	4.5	10.9	5.5	11.0	4.0	−0.05	–^a^	0.9	–	–	–	–
GAF score	74.5	12.6	73.3	13.2	75	12.4	−1.5	632	0.8	–	–	–	–

**Notes.**

a Mann–Whitney *U* test. Bolded values are *p* < 0.05; CHB, Chronic Hepatitis B; GAF, Global Assessment of Functioning; HCC, Hepatocellular Carcinoma; MINI, Mini International Neuropsychiatric Interview.

b Using married as dependent variable, while gender as independent variable after controlling for employed, personal income, current alcohol use, sleep disorder, diagnosis of HBV-related liver disease, current depression, suicidality, age, age at onset.

c Using employed as dependent variable, while gender as independent variable after controlling for married, personal income, current alcohol use, sleep disorder, diagnosis of HBV-related liver disease, current depression, suicidality, age, age at onset.

d Using personal income as dependent variable, while gender as independent variable after controlling for married, employed, current alcohol use, sleep disorder, diagnosis of HBV-related liver disease, current depression, suicidality, age, age at onset.

e Using having current alcohol use as dependent variable, while gender as independent variable after controlling for married, employed, personal income, sleep disorder, diagnosis of HBV-related liver disease, current depression, suicidality, age, age at onset.

f Using having sleep disorder as dependent variable, while gender as independent variable after controlling for married, employed, personal income, current alcohol use, diagnosis of HBV-related liver disease, current depression, suicidality, age, age at onset.

g Using having diagnosis of HBV-related liver disease as dependent variable, while gender as independent variable after controlling for married, employed, personal income, sleep disorder, current alcohol use, current depression, suicidality, age, age at onset.

h Using current depression as dependent variable, while gender as independent variable after controlling for married, employed, personal income, current alcohol use, having sleep disorder, diagnosis of HBV-related liver disease, suicidality, age, age at onset.

i Using suicidality, as dependent variable, while gender as independent variable after controlling for married, employed, personal income, current alcohol use, having sleep disorder, diagnosis of HBV-related liver disease, current depression, age, age at onset.

j Using married, employed, personal income, current alcohol use, sleep disorder, diagnosis of HBV-related liver disease, current depression, suicidality, age at onset as covariates.

k Using married, employed, personal income, current alcohol use, sleep disorder, diagnosis of HBV-related liver disease, current depression, suicidality, age as covariates.

l The *p* value of Hosmer and Lemeshow test.

m The *p* value of pearson’s chi-square test.

## Discussion

The gender ratio (452 males/182 females) in the whole sample was 2.5, supporting the notion that male predominance is consistently found in the different age groups (Baig, 2009; Yan et al., 2014). However, the male to female ratio in this study was not consistent with previous findings (Baig, 2009); Baig found that the male to female ratio was maximum in the age group of 36–40 (7.1:1) years and minimum in the age group of 51 years or above. The discrepancy across studies could be partly due to the different proportion of gender and severity of HBV-related diseases.

In this study, the proportion of patients aged 36 years and above was significantly higher than those aged 35 years or younger, which is in agreement with previous findings that the immune clearance phase in HBV-infected patients usually occurs in the age group of 20 and 35 years, and the disease progression is more likely in more advanced age (Chu et al., 1985). In addition, compared to female patients, male patients were older (48.0 *vs.* 46.9; *F* = 6.7, *p* = 0.009) and had a later onset of HBV-related diseases (34.5 *vs.* 31.9 years), which is similar to other studies (Tsay et al., 2009). Moreover, the age of onset of HBV-related diseases is influenced by biological and social factors (Lyu et al., 2016). For instance, a study conducted in China found that a history of cosmetic-related traumas and blood product use were the main risk factors of hepatitis C virus infection (Wu et al., 2012). Compared to males, females were more likely to be exposed to needle-stick procedures and blood products, especially during pregnancy, childbirth and body piercing, all of which could increase the risk of early development of HBV infections (Su & Wang, 2011).

We found that men had higher personal income and were more likely employed than women. Traditionally, men have a higher income than women in China as they are regarded as families’ economic ‘pillar’, while a considerable proportion of women prefers to stay at home as housewives. Other studies have found that women may experience more discrimination associated with HBV infections than men in China (Yu et al., 2016). Therefore, female patients with HBV-related liver diseases who suffer discrimination in China are more likely to have lower personal income and unemployment status.

In this study, the sex ratio (male/female) increased with severity of liver diseases: 1.2 in HBV carrier, 1.8 in chronic hepatitis, 3.3 in HBV-related cirrhosis and 3.9 in HBV-related HCC, suggesting that males are more likely to have severe HBV-related diseases, consistent with prior findings (Tsay et al., 2009; Wang et al., 2009). Women reported more frequent insomnia than men in this study, which is consistent with findings obtained in the general population (Zhang & Wing, 2006). Alcohol use was overwhelmingly more common in men than women in this study, which is in line with earlier findings (Xiang et al., 2009).

Some limitations should be noted. First, this is a single center study, thus the results may not be generalized to the whole country. Second, some relevant information, such as treatment and family history of HBV infections, was not recorded in this study. Third, for logistical reasons certain risk factors associated with liver diseases, such as HBV genotype or mutations, were not considered. Fourth, the ratio of male to female is unequal.

## Conclusions

A comprehensive understanding of the gender differences in demographic, physical and psychosocial characteristics among HBV-infected patients could facilitate the development of gender-specific measures for prevention, early intervention and treatment. Given the significant gender differences in HBV-related liver diseases in demographic and clinical variables, more attention should be given to clinical management according to different gender. For instance, considering the more common and severe HBV-related diseases, and alcohol use among male patients, appropriate preventive measures should be developed for male patients with HBV-related liver diseases to reduce negative outcomes.

## Supplemental Information

10.7717/peerj.13828/supp-1 Data S1 Raw data

## Additional Information and Declarations

### Data Availability

The following information was supplied regarding data availability: The raw data is available in the Supplementary File.
